# The Hepatitis C Virus-Induced Membranous Web in Liver Tissue

**DOI:** 10.3390/cells7110191

**Published:** 2018-11-01

**Authors:** Emmanuelle Blanchard, Philippe Roingeard

**Affiliations:** 1 INSERM U1259, Université de Tours & CHRU de Tours, 37032 Tours, France; emmanuelle.blanchard@univ-tours.fr; 2 Plateforme IBISA de Microscopie Electronique, Université de Tours & CHRU de Tours, 37032 Tours, France

**Keywords:** virus/cell interactions, hepatitis C virus, membranous web, liver tissue, hepatocyte

## Abstract

Host cell membrane rearrangements induced by the hepatitis C virus (HCV) have been exclusively studied in vitro*.* These studies have shown that HCV induces double-membrane vesicles (DMVs), which probably serve to separate replication sites from the cytoplasmic sensors of the innate immune response. We report for the first time the observation of HCV-induced membrane rearrangements in liver biopsy specimens from patients chronically infected with HCV. Unlike observations performed in vitro, the membranous web detected in liver tissue seems essentially made of clusters of single-membrane vesicles derived from the endoplasmic reticulum and close to lipid droplets. This suggests that the DMVs could be a hallmark of laboratory-adapted HCV strains, possibly due to their ability to achieve a high level of replication. Alternatively, the concealment of viral RNA in DMVs may be part of innate immune response mechanisms particularly developed in hepatoma cell lines cultured in vitro*.* In any case, this constitutes the first report showing the differences in the membranous web established by HCV in vitro and in vivo*.*

## 1. Introduction

Like all positive-sense RNA viruses, hepatitis C virus (HCV) induces rearrangements of the host cell membranes known as the membranous web. These membrane alterations are thought to create a platform bringing together the replicase proteins, virus genomes, and host proteins required for replication, whilst physically separating replication sites from the cytoplasmic sensors of the innate immune response [1]. The HCV membranous web was initially observed by transmission electron microscopy (TEM) on thin sections of cells expressing the entire HCV polyprotein [2], or bearing a subgenomic HCV replicon [3]. Although these initial observations revealed the presence of single-membrane vesicles induced by viral replication, subsequent studies, with a highly replicative laboratory strain (the JFH-1 virus) or its derivatives, revealed that the virus-induced membranous web consisted mostly of double-membrane vesicles (DMVs) [4,5,6]. These DMVs resembled those of other RNA viruses such as poliovirus and coronavirus. DMVs resemble small autophagosomes and the viruses inducing these intriguing organelles are known to promote autophagy, suggesting a potential link between DMV formation and the autophagic pathway [1]. The three-dimensional reconstruction of HCV-infected cell sections revealed that the DMVs were connected or tightly apposed to the endoplasmic reticulum (ER) and that only a small subset of DMVs (about 10%) had a pore-like opening to the cytoplasm [5]. This observation suggested that HCV replication occurs within the lumen of DMVs linked to the cytosol, with the pore-like connection to the cytoplasm allowing the import of all the required metabolites and the export of the newly synthesized RNA for translation or packaging into a nucleocapsid. Biochemical analyses of purified DMVs have shown these vesicles to contain an enzymatically active viral replicase capable of catalyzing de novo HCV RNA synthesis that is consistent with the hypothesis that viral replication occurs in DMVs [7]. However, replication complexes may cease to be active after the sealing of the DMV membranes [5]. 

All these data were obtained with cells cultured in vitro. The rare investigations performed on liver biopsy specimens from HCV-infected patients have not addressed the question of the membranous web, essentially describing an enlargement of the ER compartment [8] and aberrant mitochondria [9], a feature of the major stress induced by HCV in host cells. To address this question, we therefore used electron microscopy to re-examine a series of liver biopsy specimens from patients chronically infected with HCV.

## 2. Materials and Methods

We re-examined, by transmission electron microscopy, a series of liver biopsy specimens from patients chronically infected with HCV genotype 1 or 3 previously analyzed for HCV-induced steatosis [10]. Twenty-seven different liver biopsies obtained from patients with a high serum viral load (between 5.8 and 6.6 log IU/mL; mean 6.2 log IU/mL) were fully analyzed. Liver biopsy specimens were fixed by incubation for 48 h in 4% paraformaldehyde and 1% glutaraldehyde in 0.1 M phosphate buffer (pH 7.2) and postfixed by incubation for 1 h with 2% osmium tetroxide (Electron Microscopy Science, Hatfield, PA, USA). They were dehydrated in a graded series of ethanol solutions, cleared in propylene oxide, and embedded in Epon resin (Sigma, Saint-Louis, Mo, USA), which was allowed to polymerise for 48 h at 60 °C. Ultrathin sections were cut, stained with 5% uranyl acetate 5% lead citrate, and placed on electron microscopy grids coated with collodion. The sections were then observed with a Jeol 1010 transmission electron microscope (Tokyo, Japan) connected to a Gatan digital camera driven by Digital Micrograph software (Gatan, Pleasanton, CA, USA). For each biopsy specimen, at least 5 consecutive squares of the TEM grid (5000 μm^2^) were observed, representing several hundred hepatocytes.

## 3. Results and Discussion

**Figure 1:**
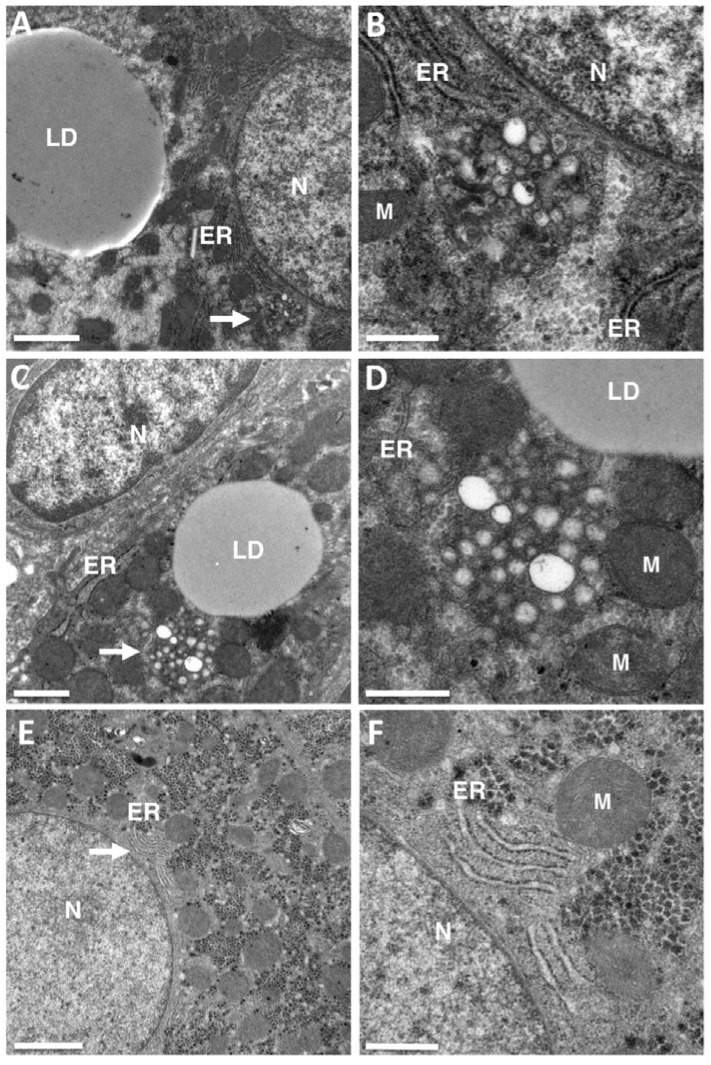
Membrane rearrangements encountered in the livers of chronic hepatitis C virus (HCV) carriers. Transmission electron microscopy (TEM) was used to re-examine liver biopsy specimens from patients chronically infected with HCV genotype 1 or 3 previously studied for morphometric analyses of the lipid droplets (LDs) accumulating in the liver during HCV-induced steatosis [10]. No double-membrane vesicles (DMVs) were detected in these liver tissues, but structures suggestive of a membranous web composed of small clusters of single-membrane vesicles were observed in several liver biopsy specimens. (**A**,**B**) Typical cluster of 10 to 30 single-membrane vesicles lying close to the endoplasmic reticulum (ER) and lipid droplets (LDs) in one patient. The area indicated by the white arrow in panel A contains a vesicle cluster characteristic of HCV-infected livers, shown at a higher magnification in panel B. This high magnification clearly shows the presence of vesicles composed of a single membrane that cannot be confused with two tightly apposed membranes. (**C**,**D**) Similar representation for a second patient. (**E**,**F**) This type of ultrastructural changes were not observed in the liver of HCV-negative patients analyzed by transmission electron microscopy in the context of other liver diseases. The area indicated by the white arrow in panel E contains normal ER, shown at high magnification in panel (**F**). N = nucleus, M = mitochondria. Bar in (**A**,**E**), 2 μm. (**C**) 1 μm. (**B**,**D**,**F**), 0.5 μm.

Despite the meticulous observation of these specimens, no DMVs were detected in this clinical material (Figure 1). The only structures suggestive of virus-induced membrane rearrangements were small clusters of 10 to 30 single-membrane vesicles lying close to the ER and the lipid droplets (LDs), which are known to be involved in the production of HCV virions. This material studied (tissues embedded in Epon by conventional means) could not be used for immuno-electron microscopy, but these clusters of single-membrane vesicles were strikingly similar to the HCV membranous web initially described in Huh7 cells harboring the con1 subgenomic replicon [3].

The discrepancies between the results of different studies may reflect properties specific to the JFH-1 strain or its derivatives which have been used in many of the investigations showing DMVs to be the most prevalent membrane rearrangements in HCV-infected cells. It has been suggested in the coronavirus model that DMVs conceal the viral RNA, enabling the virus to evade the dsRNA-triggered antiviral responses of the host [11]. Thus, the induction of numerous DMVs may be a hallmark of the laboratory-adapted JFH-1 strain, reflecting its ability to achieve particularly high levels of replication in vitro by protecting its dsRNA in sealed compartments inaccessible to the host cell dsRNA-triggered antiviral responses. However, since the early observations of Huh7 cells harboring a con1 subgenomic replicon, DMVs have been reported in such cells [5,12]. Thus, the concealment of viral RNA in DMVs may be part of the innate immune response mechanisms of the host cell itself, particularly in cell lines cultured in vitro, to limit viral replication. Alternatively, DMV formation may require levels of viral proteins achieved in cell cultures, but not in vivo. Indeed, HCV replication rates are much higher in HCV-infected cells or in cells harboring a subgenomic replicon than in vivo. HCV replication is highly sensitive to interferon and an interferon-containing environment, such as the liver, is probably less favorable to HCV replication than cultured Huh7.5 cells, which are unable to produce interferon. 

It has been suggested that HCV-induced DMVs in cell cultures are derived from single-membrane vesicles present at the early events of viral infection [6,13]. These early events observed in vitro may reflect the situation in the liver of HCV-infected patients in vivo, the subsequent induction of DMVs being a specific feature of highly replicative HCV strains propagated in cultured cell lines in vitro. We cannot exclude the possibility that DMVs are present in vivo, but in too small number for detection in liver biopsy specimens, especially since these liver biopsy specimens represent only a small part of the whole liver. However, their absence from the several hundred hepatocytes observed per biopsy specimen is remarkable, given that 7% to 20% of hepatocytes are infected with HCV in liver biopsy specimens from HCV chronic carriers [14]. The absence of DMVs from the liver biopsy specimens studied could not be explained by technical issues, as the methods used for sample fixation and preparation for transmission electron microscopy in our laboratory are the same as those used to observe DMVs in cultured cells [4,6]. Unfortunately, we were not able to confirm the presence of HCV non-structural proteins or HCV RNA in these single membrane vesicles, as these liver tissues were prepared in conventionally Epon-embedded blocks and thus precluded immuno-electron microscopy investigations. However, it should be noted that a structure very similar to that observed here was reported in the liver biopsy specimens from HCV-infected chimpanzees used for the initial description of the membranous web [2]. It remains unclear whether these discrepancies between in vitro and in vivo observations of HCV-infected cells also apply to other DMV-inducing positive-sense RNA viruses. However, this study provides the first description of the membranous web established by HCV in human liver.
